# An evaluation of the proposed organisation restructuring at Kadoma city, 2015

**DOI:** 10.11604/pamj.2017.27.20.11085

**Published:** 2017-05-08

**Authors:** A Caroline Muringazuva, Daniel Chirundu, Shepherd Shamu, Gerald Shambira, Notion Gombe, Tsitsi Juru, Donewell Bangure, Mufuta Tshimanga

**Affiliations:** 1 Department of Community Medicine, University of Zimbabwe, Zimbabwe; 2 City Health Department, Kadoma City Council, Zimbabwe

**Keywords:** Restructuring, service delivery, Kadoma city council employees

## Abstract

**Introduction:**

Restructuring is the corporate management term for the act of reorganizing the legal, operational, or other structures of a company for the purpose of making it more profitable or better organized for its present needs. However, preparing an organization to accept and welcome any change is crucial. There is concern though over poor service delivery, untimely payment of workers, top management structure which is thought to be top heavy and employee costs taking (58%) of total expenditure.

**Methods:**

A descriptive cross sectional study was carried out. A cost benefit analysis was used to assess the cost and benefits of the proposed retrenchment exercise. A descriptive cross sectional study survey was conducted to assess the workers’ perceptions towards the proposed restructuring exercise. A pretested self-administered questionnaire was used for data collection and data were analysed using EpiInfoTM (CDC 2012).Written informed consent was obtained from all study participants.

**Results:**

Sixty nine percent of the respondents were males. The median years working for the organisation was 8 years (Q1=1; Q3=17). The total income was surpassed by expenditure with USD$11 000 and 52% of expenditures was going towards employment costs. A midyear financial review showed that 1% was channeled towards capital expenditure 2% on repairs and maintenance and employee costs accounting to 58% of all incurred expenditure. Current departmental salary budget amounted to USD 3,3million dollars. Estimated salary costs for the proposed departmental structures amount to USD 3,8 million dollars. Comparison of the current and proposed structure showed that the proposed structure costs USD$486 000 more. Projected benefits of the proposed structure aims to improve service delivery from 60%-85% . Unlike managers, lower levels workers did not want the exercise to be carried out.

**Conclusion:**

The proposed structure has higher costs than the current structure but with more benefits in terms of service delivery. Generally workers perceived restructuring negatively and did not want it done.

## Introduction

Restructuring is the corporate management term for the act of reorganizing the legal, ownership, operational, or other structures of a company for the purpose of making it more profitable, or better organized for its present needs. However, preparing an organization to accept and welcome any change is crucial. A number of organizations have implemented restructuring initiativesand this is usually done after there has been a change in the operating environment [1]. For example in Zimbabwe’s managed care in the private insurance sector were Psmas and Cimas Medical Aids becoming not only financiers of care, but providers of care as well. However, preparing an organization to accept and welcome any change is crucial. Anorganisational restructuring exercise is difficult if staff are uninformed or do not see the necessity for the restructuring for they are likely to be obstructive [2].

**Figure 1:**
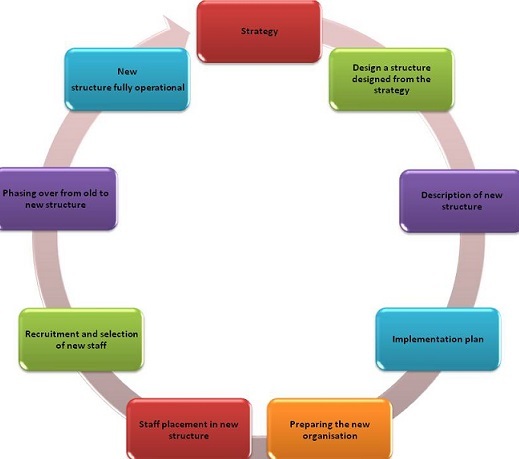
Restructuring steps

The typical preparation takes place over a period of time, and starts when staff becomes aware of the fact that management is reconsidering strategy and structure [3]. It then moves into a phase when staff is increasingly exposed to elements of the new approach. The uncertainty and turbulence of this period is not necessarily a bad thing; a degree of tension tends to operate in favour of management when it becomes clear that the new structure will address a number of difficulties and issues that staff members have been aware of. Several steps are considered in the restructuring process and StratFacdescribed the following steps in the restructuring process.Restructuring Process Steps are shown in Figure 1 [4].

A restructuring exercise is a huge undertaking, and the top management group will find it totally impossible to carry out all the required actions themselves. It is equally important that a definition of responsibilities include a clear indication of who is allowed to take which decision during the course of the process. Preparing the organization to accept and welcome the changes is crucial as the preparation takes place over a period of time. The important point about the preparation phase is that management should be as open as possible with staff.Staff replacement in new structure is the most difficult part in the restructuring process. Employees typically feel insecure under conditions of uncertainty, and tend to resist any attempt at change. Often the new structure will require new and additional skills and capabilities on a permanent basis. This briefing in turn requires that the necessary structure descriptions are available and have been approved [5].

Appropriate skills, strategies and tactics are needed in implementing and managing restructuring and retrenchment. This is partly because restructuring implies organizational change and change is normally resisted. Biller (1980) highlights some of the tactics used in carrying out retrenchment as: indicating that there are no losers or winners not favouritism for special categories of people who will win all the time, advancing general reasons for cutbacks (that are difficult to challenge, preference of some across–the-board cuts that increase fairness and legitimacy, concentrating on incentives especially for those who remain; having open minded management which is innovative, and involving customers in the search for ideas and problems to solutions. In short, having a corporate strategy is important for successful implementation of retrenchment [3].

Methods of evaluation of restructuring impact on company’s financial results should be analysed. Such analysis is important as attention to restructuring is growing; however scientific literature does not present a single methodology for an assessment of restructuring impact on value of a company and its performance results. There are analysed following valuation methods: traditional indicators of performance measurement, cash flow methods, value based methods, and cash flow return on investment. It was found that most of methods refer to profit (use accounting data) or cash flow (are based on forecasts, therefore often difficult to calculate and unreliable). The most suitable to estimate impact of restructuring on value of a company and its performance results are financial ratios and one of value based methods [6].

## Methods

We carried out a Cost Analysis of the current and proposed structures and a cross sectional survey on workers´ perceptions towards the proposed restructuring exercise at Kadoma City Council. Data were collected using a pretested, self-administered questionnaire. The questionnaire had close ended and open ended questions. For clarifications Directors were contacted. Perceptions on restructuring were measured on a five point likert scale from strongly agree to strongly disagree. Data were entered into Epi Info 7.1.5 CDC 2014. The Epi Info software was used to analyze quantitative data. Descriptive statistics were summarised using means, proportions and frequencies. Non numeric variables were re-coded to numeric for correlation analysis. Permission to carry out the study was sought and granted by the Kadoma City Council Institutional Review Board and Health Studies Office. The questionnaire had no names thus no personal information was accessed in this study. Written informed consent was obtained from participants. Confidentiality was assured and maintained throughout the study.

## Results

The demographic characteristics of the respondents shows that sixty nine percent of the respondents were males (Table 1). The median years working for the organisation was 8 years (Q1=0; Q3=17).

**Table 1:** Demographic characteristics of respondents, Kadoma city council employees, Zimbabwe 2015 (n = 68)

Variable	Subcategory	Proportion n (%)
Sex	Female	21(31%)
	Male	47(69%)
Median Age		33 (Q_1_=32;Q_3_=49)
Median years working at Kadoma City Council		8 (Q_1_=0;Q_3_=17)
Department	Central Admin	25(37%)
	Engineering	12(18%)
	Finance	12(18%)
	Health	12(18%)
	Housing	7(11%)
Grade	A	10(15%)
	B	21(31%)
	C	26(38%)
	D	4(6%)
	E	7(11%)
Status of Employment	Permanent	42(62%)
	Contract	26(38%)
Reported Level of Education	Primary	3(4%)
	Secondary	11(16%)
	Certificate	10(15%)
	Diploma	23(34%)
	Degree	21(31%)

### A financial review of the current structure, Kadoma City 2015

The budgeted income for the year was $9,013,428.00 and the expenditure was $9,002,183.23.The budgeted surplus or deficit for the year was $11, 244.77. The Kadoma City 2014 budget had salaries amounting to $298 997. Estimated costs and benefits of the proposed structure showed Health department proposing an establishment of 126 workers costing $118 739, whilst housing has proposed a new structure totalising to 61 workers (Table 2).

**Table 2:** Kadoma city 2014 Budget, Zimbabwe 2015

Income	Amount (USD)
Rates/Supplementary charges	2,439,120
Fees	5,603,054
Sales	298,200
Rent	153,054
Interest	14,000
Grants and donations	506,000
Total Income	$9,013,428.00
**Expenditure**	
Employee costs	4,665,894.73
General expenses	2,159,688.50
Repairs	460,000.00
Maintenance	353,100.00
Capital expenses	949,500.00
Asset replacement	69,000.00
Loans and charges	345,000.00
**Total expenditure**	9,002,183.23
Surplus/Deficit for the year	11,244.77
Add (Surplus)/Deficit b/f	4,033,964.36
(Surplus) Deficit c/f	4,022,719.59

### Comparison of the current and proposed structureand costs, Kadoma city 2015

Comparison of the current and proposed structurein terms of costs was summarised. Central administration costs for the proposed structure was $ 63 636 and for housing department was $26, 030, 38 (Table 3). A survey was conducted to assess workers perceptions on the proposed restructuring process. A total of 68 respondents participated in this study.Fifteen percent (n=10) of the respondents were in grade A, 31% grade B, 38% grade C and 11% in grade E. Sixty two percent of the respondents were permanent workers and 31% had attained a degree level of education. Fifty nine percent of the respondents (n=40) strongly agreed that restructuring was a form of punishment. Forty six percent (n=31) agreed that the process will improve service delivery. Thirty two percent (n=8) of health personnel strongly agreed that restructuring can improve service delivery whilst the same number disagreed. Fifty eight percent (n=14) of health workers agreed to restructuring as a cost cutting measure. Sixty nine percent (n=18) of workers in Grade C strongly agreed to creation of poverty to those affected (Table 4).

**Table 3:** Comparison of the current and proposed structure, Kadoma City, Zimbabwe, 2015

Department	Current Strength	Current Structure Costs	Proposed New Strength	Proposed structure Costs	Difference
Health	136	111,165.00	126	118,739,36	+7,574,36
Engineering	77	57,769.38	53	57,099,53	669 850
Central Administration	86	71,366.01	69	63,636,89	7.729.12
Housing	32	14,768.64	61	26,030,38	+11,261,74
Finance	52	43,927.64	56	57,434,20	+13,506.56
Total	383	298,997. 77	365	322,940.36	+23,942,59

**Table 4:** Perceptions towards the restructuring exercise among employees, Kadoma City, Zimbabwe, 2015

Perceptions	Strongly agree n (%)	Agree n (%)	Neutral n(%)	Disagree n(%)	Strongly disagree n(%)
Improving service delivery	16 (24%)	31 (46%)	10 (15%)	9(13%)	2(3%)
Adoption of results based management approach	12(18%)	29(43%)	12(18%)	12(18%)	3(4%)
Cost cutting measure	12(18%)	27(40%)	14(21%)	11(16%)	3(5%)
Create room for better working conditions	13(19%)	28(41%)	14(21%)	9(13%)	4(6%)
Reducing the number of employees	9(13%)	23(34%)	11(16%)	11(16%)	14(21%)
Firing excess staff	9(13%)	17(25%)	18(26%)	18(26%)	6(9%)
Firing poor performers	11(16%)	18(26%)	13(19%)	18(26%)	8(12%)
Establishment of effective management structure	16(24%)	30(44%)	6(9%)	14(21%)	2(3%)
Establishment of effective management for service delivery	13(19%)	26(38%)	9(13%)	15(22%)	5(7%)
Removing unqualified workers	2(3%)	12(18%	25(37%)	16(24%)	13(19%)
Removing old people due to retire	3(4%)	28(41%)	12(18%)	22(32%)	3(4%)
Premature layoff	26(38%)	16(24%)	9(13%)	15(22%)	2(3%)
Creation of poverty to those affected	33(49%)	17(25%)	9(13%)	8(12%)	1(1%)
Dismissal of employees for various reasons	19(28%)	11(16%)	14(21%)	18(26%)	6(9%)
Punishment	40(59%)	3(4%)	11(16%)	8(12%)	6(9%)

### Workers views towards the need to restructure

Ninety percent (n=9) of the workers in Grade A did not want the restructuring exercise to be done. In Grade E 86% (n=6) did want the exercise to be done whilst 14% (n=1) did not agree to restructuring exercise.

### Restructuring perceived as punishment by workers Kadoma City 2015

Sixty five percent of workers in Grade C strongly agreed to restructuring as punishment to workers. Fourteen percent (n=1) of workers in Grade E strongly agreed to the perception of restructuring as a form of punishment. Ten percent of workers in Grade B disagreed to it being punishment and 58% (n=4) of workers in Grade E strongly disagreed.

## Discussion

The expected income from rates, fees, sales and rental charges among others for the year were projected to be 9 million. It is from these charges that they got expenditure money to cover employee costs, general expenses and asset replacement. Employee costs cover salaries, wages, contract workers, allowances, councillor’s allowances, pension and medical aid. According to Urban and Rural Councils policy each authority should channel 30% of revenue collected towards employment costs and 70% towards service delivery [7]. In this case the city is using 52% of the income towards employment costs and the remaining 48% towards service delivery thereby compromising the mandate of the city.

Health department has proposed a new structure with an establishment of 126 workers costing $118 000. The health department is comprised of Environmental Health section, Personal Health and Fire and Rescue services. Its duties which are derived from the Public Health Act includes safeguarding against introduction of diseases, advising the Council on public health issues and doing research in health related issues [8]. The department removed irrelevant posts of 28 street cleaners, 8 pest controllers, 20 refuse collectors and 24 sanitation workers. These were replaced with groups which are not formally employed by the Council but offering service to the Council. The department then created 4 posts for data capturing clecks, Clinical officer post and 3 OI/ART staff members.

The Engineering department has the broad functions of planning and implementing all civil engineering projects and town planning. The department is faced by skills inadequacy, work duplication with other departments, old equipment and infrastructure and misaligned grading system. To curb these problems the department proposed a total of 53 posts which are summing up to $57 000. They replaced sewer blockage workers with groups like what health department adopted. This helped in creating 3 posts and this will help in improving service delivery within the department.

The Executive Mayor is the Chief Executive Officer of Council supported by Committee Chairpersons and a management team headed by the Town Cleck. Challenges noted by this department included declining investments in the city, decaying infrastructure, poor service delivery and lack of team work at all levels in the Council. To avoid duplication of work the department cut down on 17 posts.

The liability of the city rests on its financial position. The biggest challenge identified relates to finance. The revenue base is declining while costs are rising and this is making it more difficult to deliver quality services to rate payers. Water and sewerage have been the Councils main source of revenue contributing 40% of revenue combined but because of the economic hardships rate payers are not paying consistently. The department has however resorted to maintain its current structure.

## Conclusion

Employee costs were costing the council 52% of the total revenue collected. Compared to the current structure the proposed structure accrued $23, 942, 59 more costs. The proposed structure is reducing number of employees by 5% but improving on service delivery which is the mandate of the City Council. Generally workers perceived restructuring as a way of improving service delivery which they thought was currently below standard but regarded the process as punishment to the low grade workers and only to benefit high grade workers.

### What is known about this topic

- Organisations do restructuring to make the organisation more profitable and integrated;
- Appropriate skills, strategies and tactics are needed in implementing and managing restructuring and retrenchment;
- Several steps are considered in the restructuring process.

### What this study adds

- The need to involve the staff members in planning stage is crucial as evidenced by the responses of the participants in this study;
- Proposed structure can accrue more costs but improving on the mandate of an organisation;
- If there is no proper communication workers can perceive restructuring as punishment.

## Competing interests

The authors declare no competing interest.
